# Relationship of retinal arteriolar and venular diameters with coronary slow flow phenomenon

**DOI:** 10.1097/MD.0000000000046970

**Published:** 2026-01-09

**Authors:** Kader Eliz Sahin, Mesut Karatas, Emre Aydemir, Abdurrahman Bilen, Sedat Avci, Serkan Duyuler, Ibrahim Halil Inanc

**Affiliations:** a Department of Cardiology, Kocaeli City Hospital, Kocaeli, Turkey; b Department of Cardiology, Kosuyolu High Specialization Education and Research Hospital, Istanbul, Turkey; c Department of Ophthalmology, Adiyaman Education and Research Hospital, Adiyaman, Turkey; d Department of Cardiology, Bilkent City Hospital, Ankara, Turkey; e Department of Cardiology, Phoenixville Hospital - Tower Health, Phoenixville, PA.

**Keywords:** cardiovascular risk, coronary slow flow, microvascular disease, retina, retinal vascular caliber

## Abstract

Coronary slow flow (CSF) is claimed to be a systemic microvascular pathology rather than an isolated cardiac vascular pathology. We aimed to investigate the relation of CSF with retinal vascular calibers. This cross-sectional study enrolled 122 consecutive patients with CSF and 109 age- and sex-matched controls. Central retinal artery equivalent (CRAE), central retinal vein equivalent (CRVE), and artery-vein ratio were measured using Interactive Vessel Analyzer, a semi-automated retinal vascular analyzer. There were significant differences in CRAE, CRVE, and artery-vein ratio between the CSF and control groups (*P* < .001, .009, and <.001, respectively). Correlation analysis revealed statistically significant negative correlations between CRAE and left anterior descending artery-corrected TIMI frame count, circumflex-TIMI frame count, mean TIMI frame count, systolic blood pressure, diastolic blood pressure, and high-density lipoprotein cholesterol levels (*r* = −0.373, −0.390, −0.459, −0.416, −0.471, and −0.350, respectively, *P* < .001 for all), significant positive correlations of CRVE with left anterior descending artery-corrected TIMI frame count, mean TIMI frame count, smoking, and triglyceride levels (*r* = 0.341, 0.315, 0.404, and 0.447, respectively, *P* < .001 for all), and significant negative correlations of CRVE with systolic blood pressure and high-density lipoprotein cholesterol levels (*r* = −0.254 and −0.359, respectively, *P* < .001 for all). The CSF is closely associated with retinal vascular caliber, suggesting that, it is a systemic generalized microvascular phenomenon. The utility of retinal imaging for CSF screening is worth further investigation.

## 1. Introduction

Coronary slow flow (CSF) is characterized by delayed opacification of distal vessels on angiography without severe coronary artery disease. It is more common in young, middle-aged men and smokers. CSF is detected in ~1% to 7% of patients undergoing coronary angiography (CAG). More than 80% of patients with CSF have frequent recurrent chest pain, and ~20% require recurrent hospitalization.^[1]^

CSF has been associated with poor prognostic parameters, including fatal arrhythmias, acute myocardial infarction, and sudden cardiac death.^[2]^ Subclinical atherosclerosis, microvascular inflammation, vascular geometric irregularities, and endothelial and microvascular dysfunction have been suggested to be responsible for the pathogenesis of CSF.^[1]^ The coronary circulation consists mainly of non-resistive epicardial conductive vessels and small resistive vessels <400 μm in diameter.^[3]^ In the absence of significant epicardial stenosis, the pathology of these small resistant vessels in controlling myocardial blood flow is one of the most responsible factors in CSF pathogenesis. Right and left ventricular endomyocardial biopsies have revealed microvascular disease in patients with CSF. Capillary damage, endothelial degeneration, edema, and myo-intimal hypertrophy resulting in a narrower microvascular lumen and prolonged capillary and venous transit time have been reported in these studies.

However, there are conflicting results about whether CSF is an isolated cardiac vascular pathology or a systemic disorder involving vascular beds in other parts of the circulation.^[3]^ Since in vivo visualization of the systemic microcirculation is not readily applicable, imaging of the retinal microvasculature has been preferred primarily to predict systemic microvascular changes. It has been previously shown that the examination of the retina enables the detection of changes in the microvascular bed associated with cardiovascular diseases such as arterial hypertension (HT) and coronary heart disease.^[4]^ This is particularly due to the presence of microvascular formations in the retina of similar size and anatomy to the small resistive vessels of the epicardium; the mean calibers of retinal arterioles and venules are about 150 and 250 μm.^[5]^ In this respect, the retina has proven to be unique in that it allows direct visualization of the microcirculation in vivo. Optical coherence tomography (OCT), a noninvasive and practical imaging method that takes a few seconds to perform, is often used in the evaluation of retinal vascular and neural structures.^[6]^ With the images obtained from OCT, many parameters such as retinal arteriolar and venule diameters, and therefore retinal arteriole/venule diameter ratio (AVR), can be evaluated.^[7]^

In this study, we aimed to compare retinal artery and vein diameters and AVR of patients with CSF with those of demographically similar patients with normal coronary arteries and normal coronary flow (NCF).

## 2. Materials and methods

### 2.1. General data

**Figure 1.**
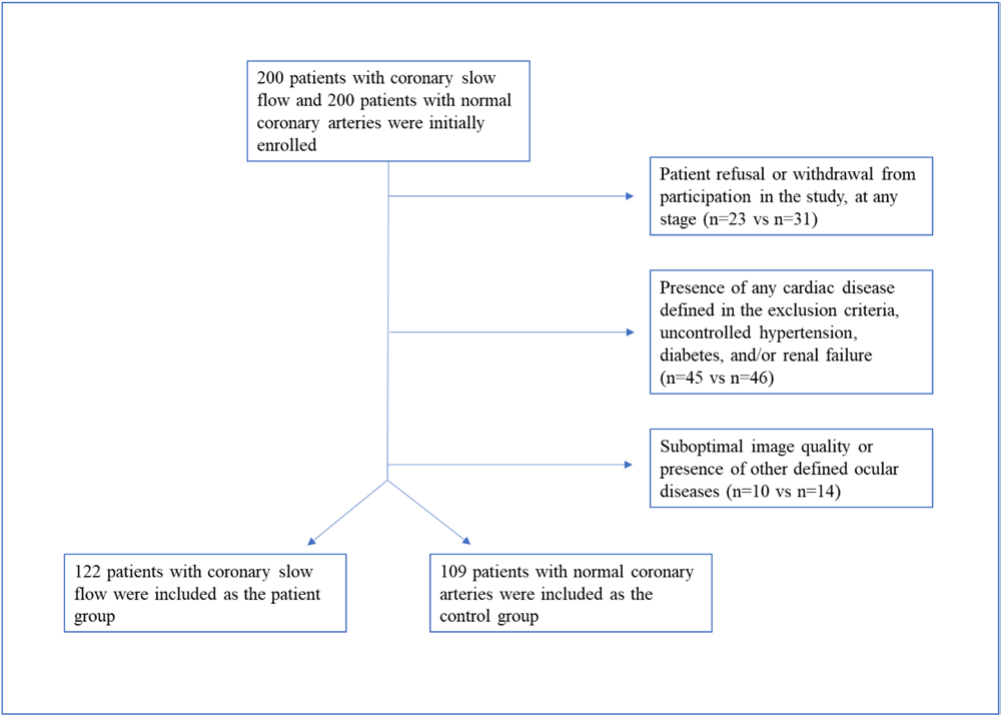
Flow diagram of patients.

Our research is designed as a cross-sectional study, and it was conducted in a tertiary referral center in Turkey between January 01, 2022 and January 01, 2023. The inclusion criteria for sampling in the study were being willing to participate in the research, being over 18 years old, and having angiographically proven CSF for the patient group and proven NCF for the control group. The sample size was calculated using Epi Info 7 for Windows (CDC, Atlanta). The calculated sample size was 96 + 96 = 192 with a type 1 error rate (ɑ) of 0.05, and a power (1 − β) of 0.95. Considering the risk of missing data and also to better represent the universe, 200 subjects in each group were included in the study. After all exclusion criteria were applied, 122 patients with CSF were assigned as the test group, and 109 patients who had completely normal coronary arteries with NCF were assigned as the control group. A flow diagram is provided in Figure 1.

Age, gender, height, weight, and body mass index (BMI) of the patients were recorded. Chronic diseases such as HT, diabetes mellitus (DM), and dyslipidemia were all considered. Blood pressure (BP) measurements were taken on bedside visits during hospitalization, and the arithmetic means of all BP measurements of each patient were recorded.

HT is defined as having BP levels of 140 mm Hg systolic blood pressure (SBP) and/or 90 mm Hg diastolic blood pressure (DBP) or taking medication for diagnosed HT, as identified in the European Society of Hypertension/European Society of Cardiology Guidelines for the Management of Arterial Hypertension.^[8]^ DM was diagnosed on the basis identified by the American Diabetes Association (2010).^[9]^ Dyslipidemia is defined according to the recommendations of the Third Report of the National Cholesterol Education Program, when elevations of any of the serum TRG (≥150 mg/dL), total cholesterol (≥200 mg/dL), low-density lipoprotein (≥100 mg/dL) levels, or reduction in serum high-density lipoprotein (HDL, <40 mg/dL in men, <50 mg/dL in women) level were present.^[10]^ Smoking was defined as regular smoking within the last 6 months.

### 2.2. Exclusion criteria

We strictly excluded CAD as well as the secondary causes of CSF, such as coronary embolism, no-reflow phenomenon, coronary ectasia, and exogenous vasoconstrictor and vasodilator administration. The other exclusion criteria were: the presence of atrial fibrillation, congenital heart disease, moderate to severe valvular heart disease, left ventricular hypertrophy, systolic heart failure, grade 2 or more diastolic dysfunction, uncontrolled stage 2 or worse HT (an average SBP ≥ 160 mm Hg and/or an average DBP ≥ 100 mm Hg), chronic obstructive pulmonary disease, cancer, glomerular filtration rate lower than 30 mL/min/1.73 m^2^, and uncontrolled diabetes (having sustained blood hemoglobin A1c levels higher than or 8%).^[11]^ Suboptimal OCT image quality and patients with a history of any chronic ocular diseases, except for refractive error, history of ocular surgery, and having an ocular abnormality (e.g., persistent fetal vasculature, optic disc hypoplasia, fovea plana) were also excluded.

### 2.3. CAG and assessment of CSF

CAG was indicated due to a positive noninvasive stress test, or rest and/or exercise angina with positive cardiac biomarkers. The left anterior descending artery (LAD) and the circumflex artery (Cx) were scanned from at least 4 views, and the right coronary artery was scanned at least from 2 views, either via right femoral or radial access. A diagnosis of CSF was made when delayed distal vessel opacification was detected in at least 1 epicardial vessel confirmed by TIMI 2 flow or TIMI frame count (TFC) > 27 (imaging at 30 frames/s).^[12]^ TFC was calculated by an experienced invasive cardiologist blinded to the data. The corrected TIMI frame count (CTFC) of LAD, which was found by dividing the CTF of LAD by 1.7, and the TFCs of Cx and right coronary artery were used to calculate the mean TFC for each patient.

### 2.4. Measurement of retinal vessel diameters

All patients who enrolled initially in the study after CAG also underwent detailed ophthalmological evaluation before discharge. Subjects with a visual acuity ≥ .8, refractive spherical equivalent ≤ 3D, and normal biomicroscopic and fundus examination were included for further evaluation. Chorioretinal parameters were evaluated using a spectral domain OCT device (Spectralis, Heidelberg, Germany) and Heidelberg Eye Explorer software (Heidelberg, Germany). The OCT evaluations were performed by a highly trained ophthalmic technician and were performed under the same dim light conditions, and a quality score ≥20 was considered acceptable. The compatibility of the results was validated by 2 experienced retinal specialists. A Visucam500 fundus camera system (Carl Zeiss Meditec, Jena, Germany) was used to take 50° colored fundus photographs. The central retinal artery and vein calibers were analyzed with the Interactive Vessel Analyzer (with the permission of Dr Nicola Ferrier at the University of Wisconsin-Madison, Madison), which is a semi-automated system that is used to conduct measurements of the retinal vessel widths via digital retinal images. The fundus photographs were taken by the same trained technician and were sent to masked, experienced researchers to measure the retinal vasculature caliber. For determination of the vascular measurement field, 3 concentric rings were placed on the fundus images, and 2 zones were described, comprising the zone extending from the disc margin to the half-disc diameter, as well as the zone extending from the half-disc to 1-disc diameter. Measurements were performed by 2 researchers separately, at different times. When the difference between the 2 measurements was more than 2%, re-measurements were performed. Inter- and intra-rater reliabilities for central retinal artery equivalent (CRAE) and central retinal vein equivalent (CRVE) measurements were assessed. In intra-rater reliability analysis, intraclass correlation coefficients were 0.99 (95% CI: 0.98–0.99) for CRAE and 0.99 (95% CI: 0.98–0.99) for CRVE. In inter-rater reliability analysis, intraclass correlation coefficients were 0.98 (95% CI: 0.95–0.99) for CRAE and 0.98 (95% CI: 0.96–0.99) for CRVE, indicating excellent agreement. The CRAE and CRVE values were calculated using the formula that was established by Hubbard et al.^[7]^

### 2.5. Echocardiography

All patients underwent TTE using the commercially available system (Vivid E9, General Electric, Horten, Norway, 2013) on admission for the CAG procedure. Standard images were obtained using a 3.5- to 5-MHz transducer from the parasternal and apical views. The TTE study was performed according to the recommendations of the European Association of Echocardiography.^[13]^

### 2.6. Blood tests

An 8- to 12-mL fasting blood sample was taken through the superficial veins of the forearm from each patient following an 8-hour fasting period. Blood samples were centrifuged at 3000 rpm for 10 minutes to separate sera. Complete blood count, high sensitivity C-reactive protein, fasting blood glucose, serum electrolytes, creatinine, and lipid panel were measured.

### 2.7. Statistical method

All analyses were performed using SPSS 17.0 (SPSS for Windows 17.0, Chicago). Continuous variables were presented as the mean ± standard deviation. Categorical variables were presented as frequencies and percentages. Kolmogorov–Smirnov test, skewness, and kurtosis were used to determine whether the continuous variables were distributed normally. Independent samples *t*-test and analysis of variance were used to compare continuous variables. The chi-square test was used to compare categorical variables. Pearson correlation tests were used to evaluate the relationship between parametric continuous variables. A point-biserial correlation was used to assess the relationship between continuous and dichotomous variables. The CRAE, CRVE, and AVR parameters were each divided into quartiles, and the CSF frequencies in each quartile were calculated using the Chi-square test. *P*-values < .05 were considered statistically significant, and an absolute correlation coefficient of ≥0.25 is considered substantial. Multivariate logistic regression analysis was performed to determine the associated factors with CSF using the enter method. The regression model was founded considering significant *P*-values in univariate analyses.

## 3. Results

One hundred twenty-two cases with SCF and 109 cases with NCF were included. Demographic and clinical characteristics of the patients are enlisted in Table 1. All continuous variables were distributed normally. The 2 groups were similar in terms of gender (with a significant male predominance in both groups), age, BMI, and prevalence of HT and DM. During the OCT evaluation, all subjects were normotensive. Smoking rate (*P* = .005), total cholesterol (*P* = .038), and triglyceride levels (*P* < .001) were statistically significantly higher in the CSF group. The mean TIMI frame counts of the subjects are enlisted in Table 2. Table 3 presents the OCT findings of the subjects where significant differences between the 2 groups in terms of CRAE, CRVE, and AVR were detected (*P* < .001, .009, and <.001, respectively).

**Table 1:** Demographic and laboratory findings of the study subjects.

	CSF (n = 122) Mean ± SD	Control (n = 109) Mean ± SD	*P*-value
Age (yr)	44.2 ± 7.6	45.3 ± 7.6	.278
Male (n, %)	103 (84.4%)	91 (83.5%)	.859
Height (cm)	171.5 ± 6.3	171.8 ± 6.2	.735
Weight (kg)	79.7 ± 4.7	79.1 ± 4.9	.335
BMI (kg/m^2^)	27.1 ± 1.4	26.8 ± 1.8	.194
Smoker (n, %)	57 (46.7%)	28 (25.7%)	.001*
SBP (mm Hg)	125.7 ± 10.2	122.3 ± 11.8	.018*
DBP (mm Hg)	78.8 ± 7.4	75.5 ± 8.4	.002*
Hypertension (n, %)	42 (34.4%)	27 (24.8%)	.109
DM (n, %)	43 (35.2%)	28 (25.7%)	.120
FBG (mg/dL)	107.9 ± 20.7	105.8 ± 21.2	.438
Total Cholesterol (mg/dL)	210.7 ± 24.6	203.5 ± 29.8	.044*
HDL (mg/dL)	39.4 ± 7.9	41.7 ± 6.0	.012*
LDL (mg/dL)	132.7 ± 21.3	126.9 ± 26.6	.068
Triglyceride (mg/dL)	193.5 ± 51.9	173.8 ± 51.3	.005*
Creatinine (mg/dL)	.94 ± .1	.95 ± .1	.760
WBC (×10^3^/L)	8.4 ± 1.7	8.2 ± 1.6	.413
Hemoglobin (g/L)	13.5 ± .8	13.8 ± 1.0	.095
Platelet (×10^3^/L)	273.3 ± 61.3	276.4 ± 63.2	.705
hs-CRP (mg/dL)	.7 ± .3	.6 ± .4	.039*
LVEF (%)	57.9 ± 6.6	57.5 ± 7.6	.678

AVR = artery-vein ratio, BMI = body mass index, CRAE = central retinal artery equivalent, CRVE = central retinal vein equivalent, CSF = coronary slow flow, DBP = diastolic blood pressure, DM = diabetes mellitus, FBG = fasting blood glucose, HDL = high-density lipoprotein, hs-CRP = high-sensitive C-reactive protein, LDL = low-density lipoprotein, LVEF = left ventricular ejection fraction, SBP = systolic blood pressure, WBC = white blood cell count.

* *P*-values < .05.

**Table 2:** Mean TIMI frame counts of coronary arteries of the study subjects.

	CSF (n = 122) Mean ± SD	Control (n = 109) Mean ± SD	*P*value
LAD TFC	39.7 ± 15.6	15.6 ± 4.5	<.001*
LAD CTFC	23.3 ± 9.2	9.2 ± 2.6	<.001*
Cx TFC	22.6 ± 9.5	12.6 ± 4.1	<.001*
RCA TFC	23.6 ± 10.4	13.5 ± 4.1	<.001*
Mean TFC	23.2 ± 4.5	11.7 ± 3.3	<.001*

AVR = artery-vein ratio, CRAE = central retinal artery equivalent, CRVE = central retinal vein equivalent, CSF = coronary slow flow, CTFC = corrected TIMI frame count, Cx = left circumflex coronary artery, LAD = left anterior descending coronary artery, RCA = right coronary artery, TFC = TIMI frame count.

* *P*-values < .05.

**Table 3:** OCT findings of the study subjects.

	CSF (n = 122) Mean ± SD	Control (n = 109) Mean ± SD	*P*-value
CRAE	172.4 ± 12.6	179.0 ± 12.6	<.001*
CRVE	218.9 ± 19.3	213.0 ± 13.6	.009*
AVR	.794 ± .094	.845 ± .090	<.001*

AVR = artery-vein ratio, CRAE = central retinal artery equivalent, CRVE = central retinal vein equivalent, CSF = coronary slow flow, OCT = optic coherence tomography.

* *P*-values < .05.

**Figure 2.**
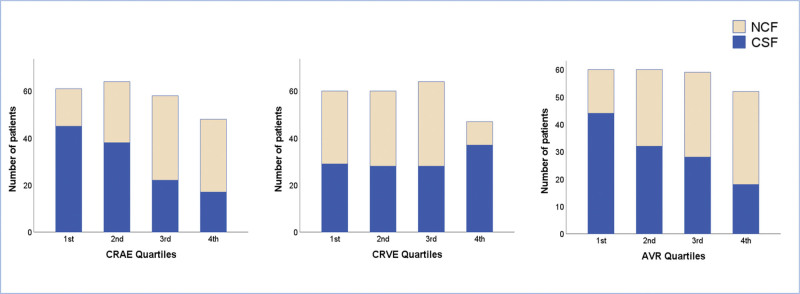
The bar chart represents the distribution of normal and coronary slow flow patients among CRAE, CRVE, and AVR quartiles. AVR = artery-vein ratio, CRAE = central retinal artery equivalent, CRVE = central retinal vein equivalent, CSF = coronary slow flow, NCF = normal coronary flow.

When we divided the OCT parameters into quartiles (CRAE to 1st: 145–165, 2nd: 166–176, 3rd: 177–186, 4th: 187–210; CRVE to 1st: 179–204, 2nd: 205–217, 3rd: 218–229, 4th: 230–255; AVR to 1st: 0.632–0.751, 2nd: 0.752–0.810, 3rd: 0.811–0.888, 4th: 0.889–1.095), patients in the highest CRVE quartile and patients in the lowest CRAE and AVR quartiles had higher incidences of CSF (37 of 47 [78.7%], 45 of 61 [73.8%], and 44 of 60 [73.3%], respectively, *P* < .001 for all) compared with other quartiles (Table 4). To better illustrate the results, a bar chart comparison of CSF incidences according to CRAE, CRVE, and AVR quartiles is shown in Figure 2.

**Table 4:** CSF frequencies according to the quartiles of CRAE, CRVE, and AVR.

	Quartiles of CRAE					Quartiles of CRVE					Quartiles of AVR				
	1st (n = 61)	2nd (n = 64)	3rd (n = 58)	4th (n = 48)	*P*-value	1st (n = 60)	2nd (n = 60)	3rd (n = 64)	4th (n = 47)	*P*-value	1st (n = 60)	2nd (n = 60)	3rd (n = 59)	4th (n = 52)	*P*-value
CSF (n, %)	45 73.8%	38 59.4%	22 37.9%	17 35.4%	<.001*	29 48.3%	28 46.7%	28 43.8%	37 78.7%	<.001*	44 73.3%	32 53.3%	28 47.5%	18 34.6%	<.001*

AVR = artery-vein ratio, CRAE = central retinal artery equivalent, CRVE = central retinal vein equivalent, CSF = coronary slow flow.

CRAE quartiles, 1st: 145–165, 2nd: 166–176, 3rd: 177–186, 3rd: 187–210.

CRVE quartiles, 1st: 179–204, 2nd: 205–217, 3rd: 218–229, 4th: 230–255.

AVR quartiles, 1st: .632–.751, 2nd: .752–.810, 3rd: .811–.888, 4th: .889–1.095.

* *P*-values < .05.

Correlation analysis revealed statistically significant but moderate negative associations of mean TIMI flow count with CRAE and AVR (*r* = −0.459 and −0.486, *P* < .001), and mild positive associations of mean TIMI flow count with CRVE, smoking, and DBP (*r* = 0.315, 0.337, and 0.268, respectively, *P* < .001 for all) (Table 5). Furthermore, we noted significant negative correlations of CRAE with LAD CTFC, Cx TFC, mean TFC, SBP, DBP, and HDL cholesterol levels (*r* = −0.373, −0.390, −0.459, −0.416, −0.471, and −0.350, respectively, *P* < .001 for all). Also, statistically significant but weak to mild positive associations of CRVE with LAD CTFC, mean TFC, smoking, and triglyceride levels (*r* = 0.341, 0.315, 0.404, and 0.447, respectively, *P* < .001 for all), and weak negative associations with SBP and HDL cholesterol levels (*r* = −0.254 and −0.359, respectively, *P* < .001 for all) were revealed. We also found significant but weak positive correlations between smoking and LAD CTFC, mean TFC, and CRVE (*r* = 0.316, 0.337, and 0.404, respectively, *P* < .001 for all). In multivariate logistic regression analysis of variables that were associated with the presence of CSF in univariate analyses, CRAE, CRVE, and AVR remained significantly associated with the presence of CSF along with smoking, HDL cholesterol, and high sensitivity C-reactive protein, while SBP, DBP, total cholesterol, and triglyceride levels lost their significant association with CSF (Table 6).

**Table 5:** Correlation analysis between TIMI frame counts of the coronary arteries and the associated risk factors.

Correlations															
		LAD CTFC	CX TFC	RCA TFC	MEAN TFC	CRAE	CRVE	AVR	Smoking	SBP	DBP	T-Chol	HDL	TRG	hs-CRP
LAD CTFC	*r*	1													
	*P*														
CX TFC	*r*	.428	1												
	*P*	<.001*													
RCA TFC	*r*	.130	.397	1											
	*P*	.048	<.001*												
MEAN TFC	*r*	.715	.813	.687	1										
	*P*	<.001*	<.001*	<.001*											
CRAE	*r*	−.373	−.390	−.253	−.459	1									
	*P*	<.001*	<.001*	<.001*	<.001*										
CRVE	*r*	.341	.220	.131	.315	−.153	1								
	*P*	<.001*	.001	.047	<.001*	.020									
AVR	*r*	−.461	−.379	−.233	−.486	.735	−.778	1							
	*P*	<.001*	<.001*	<.001*	<.001*	<.001*	<.001*								
Smoking	*r*	.316	.243	.188	.337	.163	.404	−.166	1						
	*P*	<.001*	<.001*	.004	<.001*	.013	<.001*	.012							
SBP	*r*	.152	.198	.121	.214	−.416	−.254	−.098	−.134	1					
	*P*	.021	.002	.067	.001	<.001*	<.001*	.140	.042						
DBP	*r*	.234	.205	.152	.268	−.471	−.236	−.145	−.106	.773	1				
	*P*	<.001*	.002	.021	<.001*	<.001*	<.001*	.028	.107	<.001*					
T-Chol	*r*	.150	.098	.052	.136	−.189	.121	−.203	−.028	.160	.166	1			
	*P*	.022	.139	.436	.038	.004	.066	.002	.671	.015	.012				
HDL	*r*	−.165	−.135	−.035	−.151	−.350	−.359	.017	−.325	.286	.306	.160	1		
	*P*	.012	.040	.598	.022	<.001*	<.001*	.794	<.001*	<.001*	<.001*	.015			
TRG	*r*	.220	.245	.210	.306	−.129	.447	−.389	.180	.008	−.036	.408	−.206	1	
	*P*	.001	<.001*	.001	<.001*	.050	<.001*	<.001*	.006	.908	.585	<.001*	.002		
hs-CRP	*r*	.144	.104	.006	.115	.030	−.110	.100	−.016	.088	.064	.100	.053	.063	1
	*P*	.029	.113	.931	.080	.647	.096	.131	.811	.183	.335	.130	.420	.342	

AVR = artery-vein ratio, CRAE = central retinal artery equivalent, CRVE = central retinal vein equivalent, CTFC = corrected TIMI frame count, Cx = circumflex coronary artery, DBP = diastolic blood pressure, HDL = high-density lipoprotein, hs-CRP = high-sensitive C-reactive protein, LAD = left anterior descending coronary artery, *P* = *P*-value, *r* = Pearson’s correlation coefficient (Point-biserial correlation coefficient when dichotomous variable included), RCA = right coronary artery, SBP = systolic blood pressure, T-Chol = total cholesterol, TFC = TIMI frame count, TRG = triglyceride.

* *P*-values < .05.

**Table 6:** Regression analysis for associated parameters with coronary slow flow.

	B	S.E.	Wald	OR (95% CI)	*P*-value
CRAE	−0.276	0.102	7.376	0.759 (0.622–0.926)	.007*
CRVE	0.174	0.084	4.290	1.190 (1.009–1.402)	.038*
AVR	0.046	0.021	4.613	1.047 (1.004–1.092)	.032*
SMOKING	1.106	0.371	8.908	3.022 (1.462–6.249)	.003*
SBP	−0.003	0.022	0.016	0.997 (0.954–1.042)	.899
DBP Total Cholesterol	0.055 0.007	0.033 0.006	2.832 1.162	1.056 (0.991–1.126) 1.007 (0.994–1.020)	.092 .281
HDL	−0.095	0.028	11.821	0.909 (0.861–0.960)	.001*
Triglyceride	0.002	0.004	0.338	1.002 (0.995–1.009)	.561
hs-CRP	0.869	0.402	4.677	2.384 (1.085–5.237)	.031*
Constant	−29.306	19.133	2.346	0.000	.126

*R* = 0.572, *R*^**2**^ = 0.327, *P* < .001

AVR = artery-vein ratio, BMI = body mass index, CI = confidence interval, CRAE = central retinal artery equivalent, CRVE = central retinal vein equivalent, DBP = diastolic blood pressure, HDL = high-density lipoprotein, hs-CRP = high-sensitivity C-reactive protein, OR = odds ratio, SBP = systolic blood pressure.

* *P*-values < .05.

## 4. Discussion

The present study cross-sectionally examined the association between CSF and retinal microvascular calibers. Our results demonstrated significant associations between CSF with retinal arteriolar caliber, venular caliber, and AVR.

CSF is included in the spectrum of atherosclerotic diseases, and mostly, microvascular dysfunction is involved in its pathogenesis. Strong positive correlations between retinal vascular changes and clinical predictors and outcomes of atherosclerotic heart disease have been previously demonstrated in large multicenter prospective studies.^[14]^ Multi-Ethnic Study of Atherosclerosis, which examined the relationship between cardiovascular risk factors and retinal vascular caliber, showed that decreased retinal arteriolar caliber was related to HT, whereas increased retinal venular caliber was associated with obesity, hyperlipidemia, diabetes, smoking, and systemic markers of inflammation such as CRP, fibrinogen, and interleukin-6.^[15]^ Moreover, over a 10- to 12-year follow-up period, both retinal arteriolar narrowing and retinal venule widening have been shown to predict a 40% to 70% higher risk of mortality due to coronary heart disease in middle-aged people (43–69 years).^[16]^ In the Atherosclerosis Risk in Communities study, retinal arteriolar narrowing was associated with carotid plaque as a marker of atherosclerosis and also with smoking and inflammatory markers.^[17]^ Consistent with these studies, in our study, CRAE and CRVE were both positively associated with smoking and inversely associated with BP and HDL cholesterol levels, whereas CRVE was also positively associated with triglyceride levels.

However, the significance of the associations between CSF with CRVE and AVR attenuated and was lost after further controlling for other CHD risk factors, suggesting that traditional cardiovascular risk factors are partly involved in the association. One of the involved factors is blood pressure. Increased BP, especially the diastolic component, is associated with microvascular endothelial dysfunction in resistance arteries.^[18]^ In our study, although the incidence of HT was similar between the CSF and NCF groups, there were significant differences in terms of systolic and diastolic BPs. Correlation analysis revealed weak but significant associations between SBP and DBP with CRAE and, to a lesser extent, with CRVE. The association between retinal arteriolar narrowing and increased BP is consistent with previous epidemiologic and clinical studies. These studies have shown that higher BP was associated with narrower arteriolar caliber, while its effect on venular caliber was limited. Increased BP leads to endothelial dysfunction, resulting in changes in vascular size. Endothelial function in the retinal circulation has been shown to be impaired even before clinical signs of microvascular disease in the kidneys.^[19]^ A small clinical study has shown that endothelium-dependent vasodilation of retinal arteries is impaired from the early stages of HT.^[20]^ However, we did not have data on markers of endothelial dysfunction in this report.

Lower serum HDL cholesterol was associated with wider CRAE and CRVE in our study, which is consistent with findings from recent studies.^[14–17]^ Furthermore, CRVE was associated with TRG levels. Higher TRG and lower HDL levels were also significantly associated with CSF. These findings support that the high TRG-low HDL pattern may play a role in the subclinical atherosclerotic process in both retinal and coronary vessels.

Smoking is one of the most acknowledged risk factors for CSF, along with male gender and obesity. We did not find a significant relationship between BMI and CSF. However, current cigarette smoking was significantly higher in the CSF group and also associated with larger retinal arteriolar and venular calibers. This relation has been confirmed in several studies, including Atherosclerosis Risk in Communities and Multi-Ethnic Study of Atherosclerosis.^[15,17]^ Mechanisms behind the association between smoking and larger vessel caliber remain to be elucidated, but stimulation of nitrous oxide production and potassium channel activation by nicotine and increased metalloproteinases causing fragmentation of elastic fibers and collagen might contribute to explaining the association of smoking with a wider CRAE and CRVE.^[21,22]^

CSF is a multifactorial phenomenon in which hemodynamics, as well as inflammatory status, play an important role. Concentrations of inflammatory markers such as CRP and interleukin-6 were increased in patients with CSF, and a positive association with TFC was established in a study by Li et al.^[23]^ The findings of Kalay et al also supported the results of this study.^[24]^ Meanwhile, strong associations have been shown between higher levels of inflammatory markers and larger retinal venular diameter, even this relation has led to the suggestion that retinal venular caliber might also be considered as a systemic inflammatory marker.^[15,25]^ Inflammation, which plays an important role in the pathogenesis of CSF and is strongly associated with retinal vascular changes, may also contribute to explaining the relationship between CSF and retinal vascular caliber. Regarding our sample size and the unspecified correlation of CRP with retinal vessel calibers in our results, the relationship between CSF and retinal vascular parameters cannot be explained by inflammation based on our study. However, similar to previous studies, our study showed that many factors involved in the atherosclerotic process were also associated with CRAE and CRVE. The continued significance of the association of CRVE and especially CRAE with CSF, even after controlling for those factors, increases the expectation that CSF is a systemic phenomenon.

Taha et al showed that arm-retina time and arteriovenous passage time were increased in patients with higher TIMI frame score, contributing to attest that CSF is a systemic vascular phenomenon.^[26]^ Furthermore, the demonstration of increased carotid intima-media thickness in patients with CSF also supports the concept that CSF is a manifestation of systemic atherosclerosis.^[27]^ The association of carotid intima-media thickness and CSF was significantly higher in the presence of the deletion (D) allele of the angiotensin-converting enzyme genotype. Among them, the increased frequency of the DD genotype in CSF was previously attested.^[28]^ A similar association between narrower retinal arteriolar diameter and the presence of the DD genotype was shown in the Funagata study, which examined the association of the angiotensin-converting enzyme gene polymorphism with retinal arteriolar size.^[29]^ These findings should encourage investigating the genetic contribution to the relationship between CSF and retinal vascular changes.

Another remarkable outcome of this study is that microvascular angina was associated with retinal vascular caliber, also in men. Liew et al have demonstrated that individuals with microvascular angina (formerly known as syndrome X) had similar retinal arteriolar and venular calibers as patients with coronary artery disease.^[30]^ However, after adjusting for other risk factors, microvascular angina was associated with retinal microvascular changes only in women. In our study, the male population was predominant, and we hereby report a relationship between CSF and retinal microvascular changes in a male-predominant patient group, suggesting that the retinal vessels may reflect coronary microvascular disease in men, as well. This is the first study investigating the relationship between the CSF and retinal vascular caliber measurements. Besides the association between the CSF phenomenon and retinal vessel calibers, we have also demonstrated negative correlations between TFCs and retinal arteriolar caliber and AVR, and positive correlations between TFCs and retinal venular caliber, which can be interpreted as the greater the degree of the slow flow, the easier it is to manifest systemically.

Retinal imaging, especially using OCT, holds potential as a preliminary, noninvasive screening modality in patients presenting with chest pain. These patients often represent a diagnostic challenge, as traditional imaging modalities may fail to detect underlying microvascular dysfunction. Incorporating retinal vascular caliber assessment into the initial evaluation could provide valuable insights into systemic microvascular health, allowing clinicians to identify individuals at risk for CSF or other forms of microvascular angina. The retinal vasculature, due to similarity to coronary microcirculation, offers a unique window into the systemic microvascular status. Detection of abnormal retinal vessel calibers may prompt further cardiovascular investigation, such as coronary flow reserve assessment, microvascular reactivity tests, or intensified risk factor management. Given its practicality, speed, and reproducibility, OCT-based retinal imaging may be implemented in outpatient cardiology or primary care settings. Future prospective studies are warranted to establish cutoff values, validate predictive performance, and evaluate whether retinal vascular assessments can improve patient outcomes when incorporated into routine chest pain algorithms.

## 5. Limitation

Several limitations of this study should be noted. Mainly, cross-sectional analysis limits the ability to assess the temporal sequence of reported relationships. The small sample size is another weak side of the study. Considering the presence of anginal symptoms and positive stress test in our control group, it is undeniable that some patients in the control group may also have microvascular dysfunction. This stands as a confounding factor that may reduce the strength of this study. We did neither collect data on all markers of systemic inflammation and endothelial function nor analyzed gene polymorphisms, which could further clarify the association between CSF and retinal vessel calibers. Although angiographically normal, the control group included patients with angina and positive stress test results, introducing the possibility of undiagnosed coronary microvascular dysfunction. This might have led to an underestimation of intergroup differences and should be considered in interpreting the results.

## 6. Conclusion

This is the first study demonstrating a relationship between retinal vascular caliber and CSF. Also, we confirmed the classical associations between arteriolar and venular caliber and traditional cardiovascular risk factors. Thus, this study is valuable in providing evidence that CSF is a systemic generalized microvascular disease by demonstrating the relationship between CSF and retinal vessel diameter changes, one of the systemic manifestations of atherosclerosis. However, the utility of retinal imaging for screening CSF still needs to be further investigated with broad-spectrum prospective studies.

## Acknowledgments

There is no person, institution or company to acknowledge.

## Author contributions

**Critical Review:** Kader Eliz Sahin, Mesut Karatas, Abdurrahman Bilen, Serkan Duyuler, Ibrahim Halil Inanc.

**Conceptualization:** Kader Eliz Sahin, Mesut Karatas, Emre Aydemir, Serkan Duyuler.

**Data curation:** Kader Eliz Sahin, Emre Aydemir, Abdurrahman Bilen.

**Formal analysis:** Kader Eliz Sahin, Mesut Karatas, Emre Aydemir.

**Investigation:** Kader Eliz Sahin, Mesut Karatas, Emre Aydemir, Abdurrahman Bilen, Serkan Duyuler.

**Methodology:** Kader Eliz Sahin, Emre Aydemir, Ibrahim Halil Inanc.

**Project administration:** Kader Eliz Sahin, Ibrahim Halil Inanc.

**Resources:** Kader Eliz Sahin, Emre Aydemir, Abdurrahman Bilen.

**Software:** Mesut Karatas, Serkan Duyuler.

**Supervision:** Mesut Karatas, Sedat Avci, Serkan Duyuler, Ibrahim Halil Inanc.

**Validation:** Kader Eliz Sahin, Emre Aydemir.

**Visualization:** Kader Eliz Sahin, Emre Aydemir.

**Writing – original draft:** Kader Eliz Sahin, Emre Aydemir, Sedat Avci, Serkan Duyuler.

**Writing – review & editing:** Kader Eliz Sahin, Mesut Karatas, Abdurrahman Bilen, Sedat Avci, Serkan Duyuler, Ibrahim Halil Inanc.
